# Patient Provider Continuity and Prostate Specific Antigen Testing: Impact of Continuity on Receipt of a Non-recommended Test

**DOI:** 10.3389/fmed.2021.622541

**Published:** 2021-03-19

**Authors:** Arch G. Mainous, Benjamin J. Rooks, Elvira S. Mercado, Peter J. Carek

**Affiliations:** ^1^Department of Community Health and Family Medicine, University of Florida, Gainesville, FL, United States; ^2^Department of Health Services Research Management and Policy, University of Florida, Gainesville, FL, United States

**Keywords:** continuity, prostate, value, trust, patient–doctor relationship

## Abstract

**Background:** Continuity of care with a regular physician has been associated with treatment adherence but it is unclear if continuity of care may lead to inappropriate treatments. We assessed the relationship between the receipt of prostate-specific antigen (PSA) screening, a non-recommended test, and having continuity with a single personal doctor.

**Methods:** We analyzed the 2016 and 2018 Behavioral Risk Factor Surveillance System (BRFSS). Responses from men aged 40 and older with no symptoms or family history of prostate cancer were analyzed (unweighted *n* = 232,548, representing 36,919,766 individuals). Continuity with one doctor was analyzed in relation to discussions of advantages and disadvantages of PSA tests, provider recommendation to receive a test and receipt of a PSA test.

**Results:** 39.5% of men received PSA screening during the time that the test was not recommended. Having a single personal doctor was associated with discussion of both advantages (53.3 vs. 29.7%, *p* < 0.001) and disadvantages (24.2 vs. 13.5%, *p* < 0.001) of PSA tests but also a recommendation to receive a PSA test (45.3 vs. 29.3%, *p* < 0.001). The adjusted odds of receiving a PSA test was higher among those with a single personal doctor compared to those without (OR 2.31; 95% CI, 2.17–2.46).

**Conclusion:** In a nationally representative sample during the time when PSA screening was not recommended by the US Preventive Services Taskforce, having a single personal doctor was associated with both recommendations for the test and receipt of the test. These findings emphasize the importance of the patient physician relationship and the need for evidence-based care.

## Introduction

Interpersonal continuity of care is the ongoing relationship between a patient and an individual physician. This patient physician dyad and the concomitant patient-physician relationship is a hallmark of primary care. There is evidence that having a regular physician is associated with decreased hospital admissions, emergency department visits and mortality risk (1–4).

The continuity between a patient and a physician is assumed to increase trust in the physician. This trust in the physician should therefore yield greater treatment adherence to treatments suggested by the physician. Several studies have indicated that having higher continuity is associated with greater treatment adherence in terms of medication use (5–7). There is even some evidence that continuity with a regular physician is associated with receipt of preventive services (8, 9).

Most studies focusing on continuity between a patient and a physician have centered on the receipt of needed preventive services and appropriate treatment adherence. What is unclear is an understanding of whether continuity has an impact on the receipt of low value or not universally recommended services. Low value testing is not benign and can have substantial negative impacts (10).

While differing recommendations were present based upon the specific organization, prostate specific antigen (PSA) testing was recommended by the USPSTF in 2012 as a level D, or a test that the task force recommends against (11). They concluded that there is moderate or high certainty that the test has no net benefit or that the harms outweigh the benefits. It remained as a level D recommendation until May, 2018 when it was reclassified as a C or one that is selectively offered based on professional judgement and patient preferences (12). Consequently, from 2012 until May, 2018, a PSA was a test that was recommended against by the USPSTF. Some data indicate that although prostate cancer screening significantly declined after the 2012 USPSTF guideline discouraging PSA-based screening a significant proportion of men continued to be screened (13).

The purpose of this study is to examine whether having a single personal doctor is associated with a recommendation for a test that is discouraged by the USPSTF and the receipt of such a test. Consequently, we undertook an investigation of a nationally representative survey of adult males aged 40 and older in the US to examine receipt of a PSA test.

## Methods

We analyzed the Behavioral Risk Factor Surveillance System (BRFSS) for 2016 and the first 4 months of 2018 (January–April). This time frame was chosen because it was far enough out from the last change in the USPSTF recommendations on prostate cancer screening in 2012 to ensure wide dissemination among physicians and we do not include any responses after the next change in the recommendations in May, 2018. Thus, during the time frame under study, the recommendation was a level D. The BRFSS is a nationally representative phone survey which includes both a core set of questions and optional modules that can be selected by each state or territory. Variables used in this study were obtained from the core modules of the combined landline and call phone data sets, which were sent to all 50 states, the District of Columbia, Guam, and Puerto Rico. The cohort for this study included men aged 40 and older who did not have a family history and were not symptomatic for prostate cancer so that the test was consistent with screening not case-finding.

### Prostate Specific Antigen Test

The respondents were asked if they had ever received a PSA test. Because the question was phrased as having ever received a PSA test, we classified individuals as a YES on this variable to individuals who reported that their most recent test was within the past 3 years for those queried in 2016 and past 5 years for those queried in 2018, thereby making the test within the timeframe of the level D recommendation.

### Having a Single Personal Doctor

The question on a regular source of care was asked “Do you have one person you think of as your personal doctor or health care provider?”

### Advice and Recommendation From Health Care Providers on PSA Test

Several questions were asked about advice and recommendations related to PSA testing. The respondents were asked (a) whether a doctor, nurse, or other health professional ever talked with them about the advantages of the PSA test, (b) whether they talked with them about the disadvantages of the PSA test, and (c) whether a health professional ever recommended that they have a PSA test.

### Covariates

We also included information on participant race/ethnicity, health insurance status, income, age, education, and location in the USA. Participants were classified into one of four regions in the US based off designations made by the US Census Bureau (14).

### Analysis

All analyses were conducted in R version 3.6.3, and the complex survey design of the BRFSS survey was taken into account using the survey package to allow us to make population estimates of the US state and territorial population.

Chi-square tests were computed to assess potential differences in demographics and advice and recommendations for PSA tests and having a single personal doctor between those who received a PSA test and those who did not. Chi-square tests were also computed to assess differences in advice and recommendations concerning PSA testing between those who had one regular doctor and those who don't. Finally, the relationship between having a single personal doctor and receipt of a non-recommended PSA test while controlling for the potential confounding variables of race/ethnicity, health insurance status, education, income, and region in the USA was determined using multiple logistic regression.

## Results

Of the 232,548 men surveyed in our cohort (representing 36,919,766 individuals), 39.5% of them indicated they received a PSA test during a time frame where the USPSTF recommended the procedure as a level D. Table 1 shows differences between those who did and did not receive a PSA test. Those who received PSA tests tended to be older, white, more educated, and married/cohabitating with a partner. The proportion of men who discussed either the advantages or disadvantages of PSA screening were substantially higher among those who received a PSA screening than those who did not, and discussing the advantages of the screening were twice as common as discussing the disadvantages. Similarly, the proportion of participants who reported receiving a PSA test reported that a doctor recommended they receive a PSA test at a much higher rate than those who did not receive a PSA test.

**Table 1:** Characteristics of men who did and did not receive PSA screening.

	**Received PSA Screen**	**Did Not Receive**	***P***
Total	39.5%	60.5%	
**Age**			
40–54	21.2%	70.0%	
55–69	50.9%	28.6%	
70+	27.9%	9.4%	<0.001
**Race**			
Non-Hispanic White	74.6%	63.8%	
Non-Hispanic Black	10.1%	10.3%	
Hispanic	10.2%	16.9%	
Other	5.0%	9.0%	<0.001
**Income**			
Less than $15,000	5.4%	11.3%	
$15,000–$24,999	10.7%	15.9%	
$25,000–$34,999	8.2%	10.2%	
$35,000–$49,999	13.2%	12.5%	
$50,000 or more	62.5%	50.1%	<0.001
**Education**			
High School or Less	33.4%	49.3%	
Some College	30.3%	26.8%	
College or Higher	36.3%	23.9%	<0.001
**Marital Status**			
Married/Cohabitate	75.0%	63.6%	
Never Married/ Separated/ Divorced	25.0%	36.4%	<0.001
**Region**			
West	20.8%	25.1%	
Midwest	20.8%	21.8%	
Northeast	17.1%	16.8%	
South East	39.6%	35.6%	<0.001
Have one regular doctor	86.0%	67.9%	<0.001
Discussed advantages to PSA	89.3%	20.3%	<0.001
Discussed disadvantages to PSA	40.4%	10.2%	<0.001
Doctor recommended PSA	87.5%	10.8%	<0.001

The results of Chi-square tests of independence between patients who have a single personal doctor and whether they discussed and advantages and disadvantages of PSA testing or were recommended PSA testing are shown in Table 2. Discussion of the merits of a PSA test and receiving a recommendation for a PSA test were both higher among those who reported having a single personal doctor.

**Table 2:** Discussion of PSA screening by continuity of care.

	**Have a Regular Doctor**	**No Regular Doctor**	***p***
Discussed advantages to PSA	53.3%	29.7%	<0.001
Discussed disadvantages to PSA	24.2%	13.5%	<0.001
Provider recommended Patient have a PSA	45.3%	25.3%	<0.001

The results of a logistic regression analysis on the association between having a single doctor and receiving a PSA test indicates a significant relationship even after controlling for potential confounding variables. The odds of receiving a PSA test is 2.90 (95% CI: 2.74–3.06) times higher among those with a single personal doctor than those who do not report having a single doctor. After adjustment, the odds of receiving a PSA test is 2.31 (95% CI: 2.17–2.46) times higher.

## Discussion

This study demonstrated that patients who received a non-recommended, low value preventive service (PSA testing) was strongly linked to patients having one person they think of as their personal doctor or health care provider. As noted previously, higher interpersonal continuity is associated with several factors associated with high quality of care, such as greater medication adherence and receipt of preventive services. Further, past research has shown that physician recommendations directly influences patient behavior and perceptions of risk (15, 16). This study extends the previous literature by examining recommendations and receipt of a test consistent with low quality of care and interpersonal continuity.

The finding that nearly 40% of patients received this level D recommendation test and the test was recommended by a large number of patients' regular physicians and health care providers is worrisome. A large number of patients are being tested inappropriately and a large number of physicians and health care providers are recommending a test whose results do not benefit and, in actuality, may harm these patients. In this instance, having a regular doctor appears to be associated with low value care that may lead to harmful additional testing and treatment.

The physicians ordering the test may have specific reasons for their recommendation and ordering of a low value test. One reason may be that they were unfamiliar with the USPSTF recommendations. However, our study design examined tests that were received during the time the PSA test was a level D and we limited the assessment of responses to a time frame that was at least 4 years after the recommendation was initially disseminated suggesting that the recommendation had been present in the community for a substantial amount of time. Other reasons for deviating from USPTF guideline may include personal reasons for deviating from guidelines, disagreement with guidelines and using other recommended guideline (such as the American Urological Association) in decision making. Between 2016 and 2018, the time of this study, the American Urological Association recommended no routine screening for men aged 40–54 but encouraged shared discussion between patients and physicians for men 55–69, advice not completely consistent with the USPSTF (17). Agreement with guidelines and behavior has been shown for recommended tests as well. Physician disagreement with guidelines has also been shown to be associated with the low level of screening for abnormal glucose, a USPSTF level B recommendation (18, 19).

The trust placed in a physician or other health care provider is paramount to a patient's decision-making regarding testing such as PSA. Patients will comply with recommendations from a physician. Our findings reinforce the importance of evidence-based guidelines in care. Evidence-based guidelines and standardization of practices are meant to minimize ordering of non-recommended testing. A variety of strategies have been used to try and minimize low value care. The case of imaging for low back pain provides some lessons (10, 20). For example, a best practice advisory or electronic medical record (EMR) alert is presented when a physician or other health care provider is attempting to order the test in question (21). Another possible strategy would be to restrict order placement within a health system without directly acknowledging the test is non-recommended.

An additional point that needs to be made is that these results have some implications for health equity. Even though these are low value, non-recommended tests, they are more likely to be recommended to White, high-income patients. This seems consistent with this patient population more likely to receive medical tests. The primary point here is that they are also more likely to receive inappropriate tests. Which has implications for both the distribution of healthcare resources but also the importance of evidence based medicine.

Several limitations to this study are present. Although we attempted to limit the length of time that respondents needed to remember to discuss the test, there still may have been somes recall bias. A second limitation is that we classified individuals who reported having more than one doctor as not having a regular doctor. It is possible that one physician among several seen by the patient may have had a strong continuous relationship with the patient. Third, we do not know the motivation or the knowledge base of the providers who recommended the tests. Fourth, it is possible that the patients suffer from hypochondriasis and pressured the physicians to order the test. This is possible but the prevalence of hypochondriasis is ~5% and so this may have played a role but the prevalence is so low that it cannot explain the primary findings.

In conclusion, the results do not suggest that interpersonal continuity with one regular doctor is not important. Quite the contrary, these results reinforce the power of that relationship but the results also point to the impact, in a negative way, if the regular doctor does not follow evidence-based guideline consistent care. The patient physician relationship and trust in one's physician is critical in providing care but the concomitant responsibility that falls to physicians is to provide the best high quality care.

## Data Availability Statement

The original contributions presented in the study are included in the article/supplementary material, further inquiries can be directed to the corresponding author/s.

## Author Contributions

AM, BR, and PC: conceptualization. AM and BR: data analysis. AM, BR, EM, and PC: drafting of manuscript. All authors participated in the project and reviewed and approved the final submission.

## Conflict of Interest

The authors declare that the research was conducted in the absence of any commercial or financial relationships that could be construed as a potential conflict of interest.
